# A Model for Online Delivery of Multiple Mini Interviews

**DOI:** 10.1177/23821205231183875

**Published:** 2023-06-20

**Authors:** Simon C Cork

**Affiliations:** 1 School of Medicine, 2369 Anglia Ruskin University, Chelmsford, UK

**Keywords:** medical school, admissions, COVID-19, multiple mini-interviews, online interviews

## Abstract

Multiple mini interviews (MMIs) have become the mainstay of medical school admission interviews in the United Kingdom. During the COVID-19 pandemic, Government imposed restrictions on the meeting of people indoors precipitated a move towards conducting interviews online. Thus, the development of methodologies to conduct robust MMI style interviews remotely was required. In this article, a validated method for conducting remote MMIs is described. This method of delivery produced comparable candidate scores compared with pre-pandemic in-person interviews and maintained reliability.

## Introduction

The global COVID-19 pandemic led to an unprecedented shift to remote online working in almost all aspects of life. Although successful vaccination programs to control the spread and effects of the virus have led to a return to some level of normality, certain aspects of the move to online working are likely to remain long term. During the pandemic, university admissions processes continued, albeit adopting this shift towards conducting interviews remotely. Many medical schools in the United Kingdom have adopted the method of multiple mini interviews (MMIs) to conduct their pre-offer interviews and studies have shown that this method provides many advantages to candidate selection over traditional panel interviews, including increased reliability and validity.^1‐3^

The shift to remote delivery of interviews introduced significant challenges to their delivery, not least the lack of available examples on which to model their approach. Nevertheless, the continuation of online interviews in the “post-pandemic” era offers tangible benefits, including equitable access for those for whom traveling to a university for an in-person interview may be cost prohibitive, or who may be applying from overseas.^4^ Here a method of successfully delivering online MMIs to pre-offer medical school candidates is described. This model may be useful for other universities in planning the delivery of their interviews for the future.

### Anglia Ruskin University model

Anglia Ruskin University (ARU) School of Medicine, located in Chelmsford, Essex, England opened in 2018, with an annual intake of 100 students onto the undergraduate medical program. Interviews conducted during the 2019/2020 interview period (which took place in December 2019 and January 2020) were conducted in person. Following government guidelines on the gathering of people indoors during the COVID19 pandemic, which were in place from March 2020, the 2020/2021 interview period (which took place in December 2020 and January 2021) took place remotely. Analysis of the data included in this report was approved by the ethics panel of the Department of Allied Health, Nursing & Midwifery and Medicine ethics board at ARU (ETH2223-3583). As this was a retrospective analysis, informed consent from participants was not required.

## Platform

Remote interviews were conducted using Zoom (Zoom Video Communications, San Jose, California). This platform allows all participants to be present in one area, as well as providing the options for multiple separate “break-out” rooms. During the remote delivery, a Microsoft Teams chat was running concurrently, accessible by support staff to monitor any technical issues that may be ongoing in any of the stations.

Prior to the interviews, assessors and candidates received instructions on how to access the platform, with assessors provided additional guidance on how to access the scoring platform, as well as processes for what to do in the event of technical issues.

## Staffing requirements

The delivery of this model of remote MMI necessitates the addition of support staff. A total of 9 support staff were required to administer and provide technical support across the online 6-station MMI. This compares with 3 support staff required to administer in-person interviews. Table 1 describes the administrative support required and their respective roles.

**Table 1.** Outline of roles and responsibilities for administrative staff during remote delivery MMIs.

STAFF (NUMBER REQUIRED)	ROLE
Lead (x1)	Managerial oversight of the process Welcome candidates Monitor concurrent Microsoft Teams chat
Deputy lead (x1)	Monitor concurrent Microsoft Teams chat Move candidates between rooms Provide timings and instructions for when to start and end each station
Backup (x1)	Emergency assessor/role player/technical support if connection was lost by one of those members
Technical support (x6)^a^	Communicate with lead/deputy lead to alert in case of technical issues in respective station Display station instructions to candidates

^a^ Note that technical support staff were not required for the in-person delivery of MMIs. Technical support was comprised of administrative staff within the School of Medicine, as well as students temporarily employed for the duration of the admissions period.

## Total number of interviews

During the 2020/2021 interview round, a total of 595 candidates were interviewed. ARU School of Medicine uses the University Clinical Aptitude Test^5^ in order to rank candidates for interview. Candidates invited to interview during the 2019/2020 and 2020/2021 interview period were those candidates who scored in the top 600 of total applicants that year. The number of stations was reduced compared to the in-person 2019/2020 interview run (6 × 7 min stations in 2020/2021 compared to 8 × 7 min stations in 2019/2020), however the number of candidates interviewed remained comparable (595 candidates in 2020/2021 compared with 593 candidates in 2019/2020). Due to the reduced number of stations, the total number of individual interviews was therefore less in 2020/2021 compared to the previous year (3570 in 2020/2021 compared with 4744 in 2019/2020).

### Mode of delivery

#### Assessors

**Figure 1.**
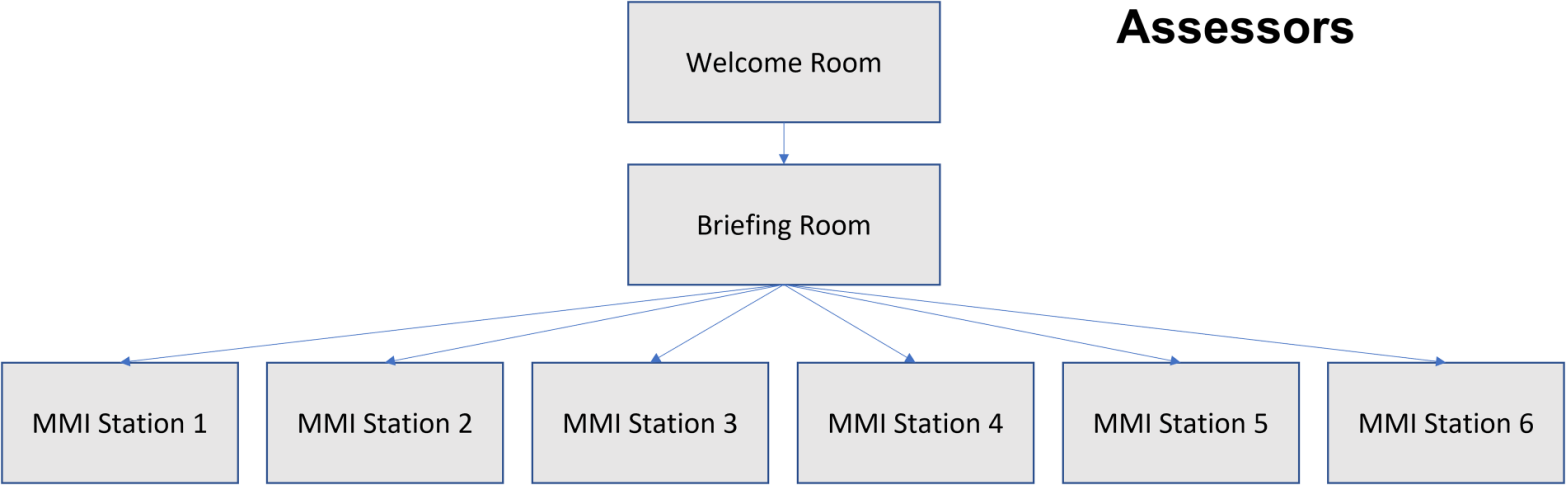
The journey of the assessor through the online MMI run. Assessors joined into an initial “Welcome Room” where their identity and station was confirmed. Assessors were then transferred into a “Briefing Room” where the admissions administrator instructed assessors on how the day would run and conduct microphone and camera checks. Assessors were then moved into their respective MMI station breakout rooms.

All interviews and pre-interview sessions were held on Zoom. A pre-interview briefing was held prior to the interview day with all assessors to explain the method of delivery and to answer any queries. On the day of interview, assessors joined the session through an invited link, into which they joined a “Welcome Room” (Figure 1). After confirming identity and correct session, assessors were moved into a “Briefing Room.” Each assessor was ascribed an MMI station, which constituted a single breakout room in Zoom into which they were moved. Each MMI station was supported by a technical support (a member of the School administrative team), whose role was to display the MMI station scenario or question at the start, to provide technical assistance if required, or to temporarily step into the role of assessor if the main assessor experienced connection issues. Certain MMI stations which employed a role-play scenario were also joined by a role player, who was a member of our patient partners program. Individual assessors, technical support, role players and candidates were moved by 2 members of staff who sat outside of the MMI stations. Assessors were booked for an entire day with 15-min breaks between runs.

#### Candidates

**Figure 2.**
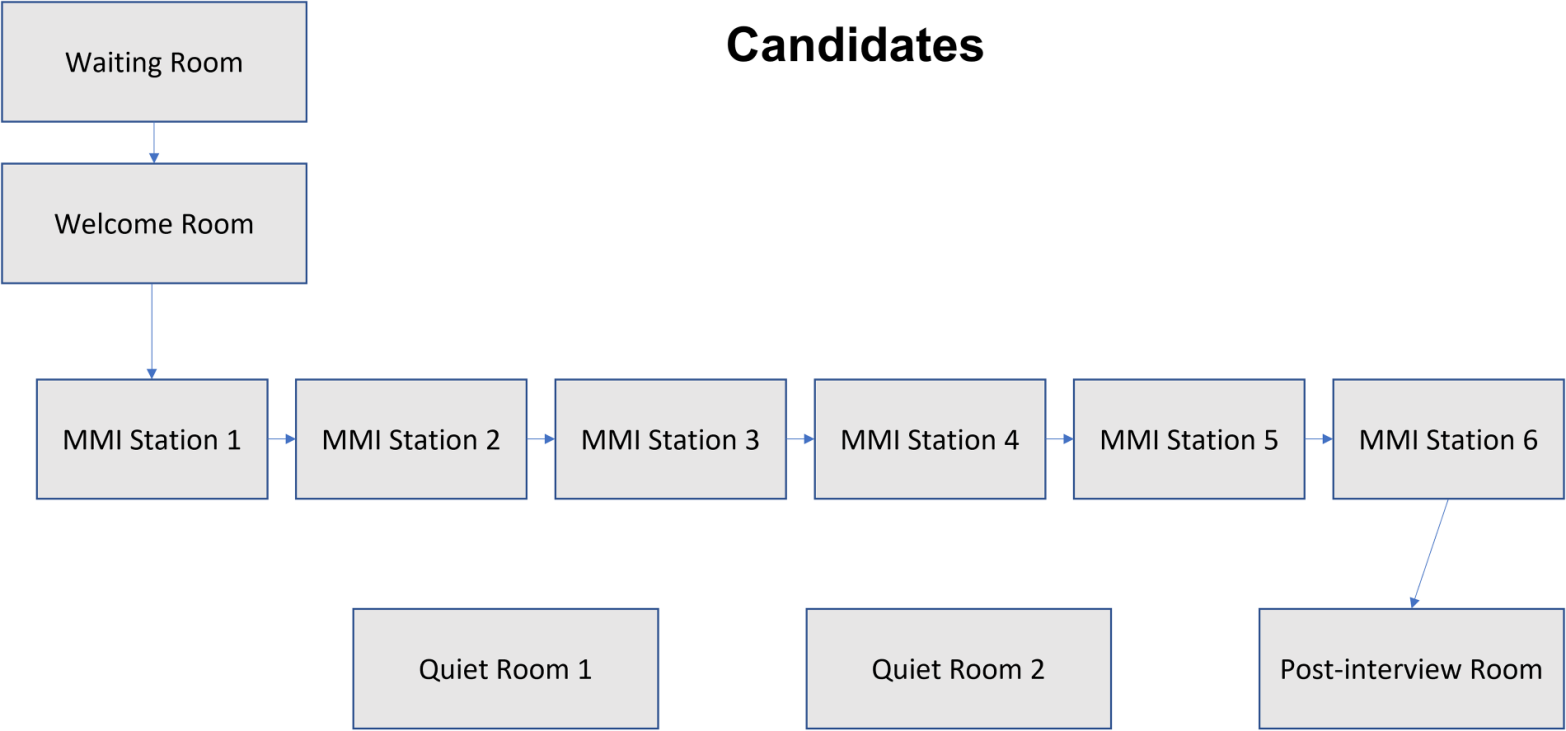
The journey of candidates through the online MMI run. Candidates joined a waiting room before being transferred into a Welcome Room, where candidates were briefed, as well as having their identification and connectivity checked. Candidates then joined 1 of 6 MMI stations, which were set up as breakout rooms and cycled between breakout rooms until all 6 stations had been completed. At the end of the run, candidates were moved to a post-interview room where members of the admissions team and student representatives were available to answer any questions. Two quiet rooms were made available for any candidates who become distressed or needed a break during the interview cycle.

Candidates were instructed to attend at a set time and were provided with a Zoom link specific for their session. When candidates joined the session, they were initially held in a waiting room before being invited individually to a Welcome Room (Figure 2), hosted by the admissions lead. Here identification and connectivity were checked. Candidates were then moved to an Admissions Room for a pre-interview briefing which instructed them on the format of the interview. Candidates were asked to change their Zoom name to their candidate number, surname, and initial.

At the stated start time, candidates were moved to their first MMI station through use of the “breakout room” function in Zoom. Once all candidates were in their station, a message was displayed instructing the interview to begin. After 7 min had elapsed, a further message was displayed instructing the interview to stop. Candidates were then moved to their next station by the admissions lead. At the end of all 7 stations, candidates were moved to a post-interview room where they were met by the admissions team and student representatives.

Further breakout rooms were made available for any candidates who experienced emotional distress during the interview process and who needed to be moved out of the interview round. Here the admissions team would meet with the candidate to enquire whether they felt they were able to continue.

#### Changes to interview stations

In-person interviews conducted in our medical school take 3 main forms: question based (eg, investigating a candidates motivation to study medicine), task based, and role play. Although the move to online interviews meant many of the stations previously used for in-person interviews could be used unchanged, some tasks were either not suited to this delivery or had to be modified.

A role play station, in which candidates had to reorder a list of priorities prior to explaining their reasoning to an actor was modified to reduce the number of priorities. This allowed candidates time to write down the list if they wished, in contrast to in-person interviews where they receive a printed version of the priority list.

Online delivery of interviews also prevents the use of task-based interview stations, where candidates or actors must physically interact with something in the interview station. Such tasks are designed to test a candidate's ability to communicate clearly and adjust their communication in response to confusion or anxiety by the actor. Although these stations are useful, in as much as they add an element of interactivity to the interview and are more difficult for candidates to produce a scripted response, the elements that they test can be achieved through standard role-playing stations.

During in-person interviews, candidates stand outside of a booth for 1 min to allow them to read the instructions for that station. During online interviews, candidates were moved to the respective stations breakout room where the instructions were displayed for 1 min, after which the instructions were removed, and the interview commenced.

No changes were made to the scoring proforma. Assessors scores were inputted into the same software that is used for in-person interviews. A contingency Microsoft Excel scoring sheet was made available to all assessors in case of any technical issues with the software, however this was not required.

## Reliability

To assess whether conducting interviews online was as reliable as conducting face-to-face, mean applicant score and station reliability (as calculated using Cronbach's alpha) were compared against previously conducted, face-to-face MMIs (run during the 2019/2020 interview round). Both mean applicant score, and Cronbach's alpha were comparable between the 2 interview rounds (Table 2), suggesting the delivery of MMIs online did not compromise the reliability of the interview process.

**Table 2.** Mean applicant scores and Cronbach's alpha score for online MMIs compared with face-to-face (held during the 2019/2020 interview round).

MODE OF DELIVERY	MEAN APPLICANT SCORE (± SE)	CRONBACH'S ALPHA
Face-to-face	74.29 ± 0.389	0.696
Online	75.74 ± 0.388	0.625

## Technical issues

Over the course of 15 interview days there were a total of 58 candidate connection issues and 29 assessor, role player or room support connection issues. In the case of connectivity issues with the assessor/role player, the technical support in that respective station alerted the lead/deputy lead, at which point either the technical support or back up support staff were brought in to take over.

The response to a loss of candidate's connectivity depended on the length of time it required for the candidate to re-join the call. If a candidate was able to successfully re-join within 7 min, they continued in the scheduled station with the remaining time available. If a candidate was unable to re-join within 7 min, they were not able to complete the remaining stations. In this instance, if the candidate had missed 1 or 2 stations, then the average score across the completed stations was applied. If a candidate missed 3 or more stations, they were offered the opportunity to interview for the missed stations only at a later date.

Over the course of the interview period, 8 candidates had average scores applied to 1 station and 1 candidate had average scores applied to 2 stations. The remaining candidates who experienced connectivity issues were able to re-join and complete all stations.

## Discussion

Here a successfully delivered online MMI for medical school applicants during the 2020/2021 interview round is described. This format can act as a model for other universities and medical schools to implement a similar style of interview format in the future. Whether delivering interviews remotely provides substantive benefits over traditional face-to-face interviews will be dependent on the circumstances of the individual medical school and candidates. Perhaps the main benefit is in costs incurred to the candidate, who do not have to travel to individual campuses to conduct their interviews. Indeed, research has shown that the shift to online delivery of interviews for a pharmacy course resulted in a significant uplift in the number of applicants from underrepresented students,^6^ demonstrating that the provision of online interviews has the potential to remove barriers to candidates that might otherwise be marginalized. However, such interviews are often combined with campus tours, which may be more difficult to implement virtually.

This method is not without difficulties, including the significantly enhanced technical support that is required to monitor and administer multiple Zoom calls at once. This method is also subject to technical disruption, such as connectivity issues, which lead to a small minority of students unable to complete the entire interview process and having average scores applied.

Despite these challenges, the model described demonstrates that the shift to online interviews can be achieved without compromising the reliability of the process. A limitation of this report is the narrow duration of the study period. Due to the nature of the interview process, this report was unable to influence the number of subjects included in the data analysis. Analysis of a greater number of interviews held in both online and face to face format would strengthen the analysis of this model's reliability. Nevertheless, the model described here should provide medical schools with confidence that remote delivery of interviews can be achieved without compromising on the robustness of the admissions process.

## Conclusion

The report demonstrates an effective method for the delivery of online MMIs for the selection of medical school candidates. The demonstrated maintenance of reliability compared with face-to-face MMIs should provide confidence to admissions leads that this method does not compromise the integrity of the interview process.
